# Pharmacists’ Perspectives of Their Roles in Antimicrobial Stewardship: A Qualitative Study among Hospital Pharmacists in Malaysia

**DOI:** 10.3390/antibiotics11020219

**Published:** 2022-02-09

**Authors:** Wan Mae Lai, Farida Hanim Islahudin, Rahela Ambaras Khan, Wei Wen Chong

**Affiliations:** 1 Centre of Quality Management of Medicines, Faculty of Pharmacy, Universiti Kebangsaan Malaysia, Kuala Lumpur 50300, Malaysia; laiwanmae@yahoo.com (W.M.L.); faridaislahudin@ukm.edu.my (F.H.I.); 2 Pharmacy Department, Serdang Hospital, Ministry of Health, Kajang 43000, Malaysia; 3 Pharmacy Department, Kuala Lumpur Hospital, Ministry of Health, Kuala Lumpur 50586, Malaysia; rahela.ak@gmail.com

**Keywords:** antimicrobial stewardship, antimicrobial resistance, pharmacist, roles, barriers, facilitators

## Abstract

Antimicrobial resistance has negatively impacted patient outcomes and increased healthcare costs. Antimicrobial stewardship (AMS) includes all activities and policies to promote the judicious use of antimicrobials. Pharmacists are key players in AMS models worldwide. However, there is a research gap in the role of pharmacists as antimicrobial stewards in Malaysia. This study aimed to explore hospital pharmacists’ perspectives on their roles in, and barriers and facilitators to the implementation of AMS strategies. Individual, semi-structured interviews were conducted with 16 hospital pharmacists involved in AMS activities from 13 public hospitals in Kuala Lumpur and Selangor. Audio-taped interviews were transcribed verbatim and imported into NVivo software version 10.0 (QSR). A thematic analysis method was used to identify themes from the qualitative data until theme saturation was reached. Respondents perceived pharmacists as having important roles in the implementation of AMS strategies, in view of the multiple tasks they were entrusted with. They described their functions as antimicrobial advisors, antimicrobial guardians and liaison personnel. The lack of resources in terms of training, manpower and facilities, as well as attitudinal challenges, were some barriers identified by the respondents. Administrative support, commitment and perseverance were found to be facilitators to the role of pharmacists in AMS. In conclusion, pharmacists in public hospitals play important roles in AMS teams. This study has provided insights into the support that AMS pharmacists in public hospitals require to overcome the barriers they face and to enhance their roles in the implementation of AMS strategies.

## 1. Introduction

Antimicrobial resistance (AMR) has been plaguing us in the last two decades [1,2]. Multiple studies have shown that those with infections caused by resistant microorganisms are associated with increased morbidity and mortality [3,4,5,6]. A similar trend was observed in Malaysia, where carbapenem-resistant *Enterobacteriaceae* cases increased exponentially [7]. Inappropriate prescribing and overuse of antimicrobials among humans, as well as extensive agricultural use, have been identified as the main driving forces towards AMR [8].

Activities and policies aimed at improving the judicious use of antimicrobials, collectively known as antimicrobial stewardship (AMS), have shown some success in tackling the overuse and inappropriate prescribing of antimicrobials [2,8]. The pivotal role of pharmacists in promoting judicious antimicrobial prescribing has been recognized by many international reports [1,9,10]. This has led to the inclusion of pharmacists as key players in multi-disciplinary AMS models developed across many countries [11,12,13,14,15].

AMS was formally introduced in Malaysia with the publication of the Protocol on Antimicrobial Stewardship Program in Healthcare Facilities, in 2014 [16]. The protocol spells out the general functions of an AMS team as well as the roles of individual AMS team members. Similar to other AMS models worldwide, diverse roles and responsibilities have been entrusted to pharmacists, including ensuring the safe and effective use of antimicrobials, surveillance of antimicrobial use, audit and feedback, and enforcing an approval system of restricted antimicrobials [16].

Variations in AMS practices among the public hospitals in Malaysia are expected, given the differences among hospital categories in terms of specialties, manpower and facilities. These differences may affect pharmacists’ roles in the implementation of AMS strategies. It is important to know the actual practice and roles of pharmacists in the implementation of AMS strategies in Malaysian public hospitals, and the barriers they face, in order to facilitate future role improvement and expansion. There is limited qualitative research that has been conducted in Malaysia to explore pharmacists’ perceptions of their roles as antimicrobial stewards. This study aimed to explore hospital pharmacists’ perspectives on their roles in, as well as barriers and facilitators to the implementation of AMS strategies.

## 2. Materials and Methods

This qualitative study involved individual semi-structured interviews with hospital pharmacists who had been elected as AMS team members in their workplace, to elicit their views and perspectives on the roles they play in the implementation of AMS strategies. Purposive sampling was used to select at least one AMS pharmacist from each public hospital in the Federal Territory of Kuala Lumpur and the state of Selangor.

### 2.1. Data Collection and Sample

An interview guide was developed for this purpose using domains identified through a literature review [17,18,19,20,21,22]. The individual interviews between the researcher and the respondent were conducted from March to July 2018, at the respondent’s preferred location; either at the respondent’s workplace or via telephone. All interviews conducted were audio-recorded with the consent of the respondents. The audio recordings of the interviews were then transcribed verbatim. The transcripts were emailed to the respondents to obtain any additional comments. Each of the transcripts was assigned a code and potentially identifying information was removed.

### 2.2. Data Analysis

Transcripts were imported into NVivo software version 10.0 (QSR) to facilitate a systematic coding process to identify themes from the qualitative data, using a thematic analysis method. Codes were assigned to the interview transcripts, further refined and grouped into categories that represent the main themes emerging from the data. The transcripts were initially broadly coded to generate a preliminary coding system. New codes were developed subsequently as new themes began to emerge. Emerging themes and sub-themes were continually refined, tested and revised using a constant comparison approach. The data were evaluated until theme saturation was reached. All analyses were facilitated by discussions with co-researchers to refine and clarify existing themes, until a consensus was achieved.

## 3. Results

A total of 16 practicing AMS pharmacists from 13 public hospitals were interviewed for this study. Out of the 16 pharmacists interviewed, one was from a university-affiliated hospital, two were from state hospitals, nine were from major specialist hospitals, one was from a minor specialist hospital and three were from non-specialist hospitals. The profiles of the respondents are summarized in Table 1.

Findings related to the pharmacists’ roles, and the barriers and facilitators, are described below, along with illustrative quotations for the themes and sub-themes identified.

### 3.1. Pharmacists’ Roles in the Implementation of AMS Strategies

All respondents were of the same general opinion that AMR is an issue of increasing concern and most perceived this to be a common issue in their setting. They perceived pharmacists as having important roles in the implementation of AMS strategies because of their responsibilities in the procurement and supply of antimicrobials, as well as their ability to draw on their knowledge and expertise to promote the judicious use of antimicrobials:

*“Pharmacists do play very important roles because we know [the] stock levels (of antibiotics), contributing factors to high usage of carbapenem and [other antibiotics], and we know the cost of each antibiotic, we know the appropriate dosing, pharmacokinetics or pharmacodynamics effects, and when it’s not justified, what to recommend, what other alternatives we have, and whether any dose adjustment [is] needed… So definitely, pharmacists [are] very important, no one can replace [them]…”*

(Respondent 14, university/state/major specialist hospital, 2 years of experience in AMS)

The sub-themes on the role of pharmacists encompass (i) pharmacists’ diverse functions, (ii) pharmacists’ visions on role expansion and (iii) the impact of pharmacists’ involvement in AMS.

#### 3.1.1. Pharmacists’ Diverse Functions

**Figure 1:**
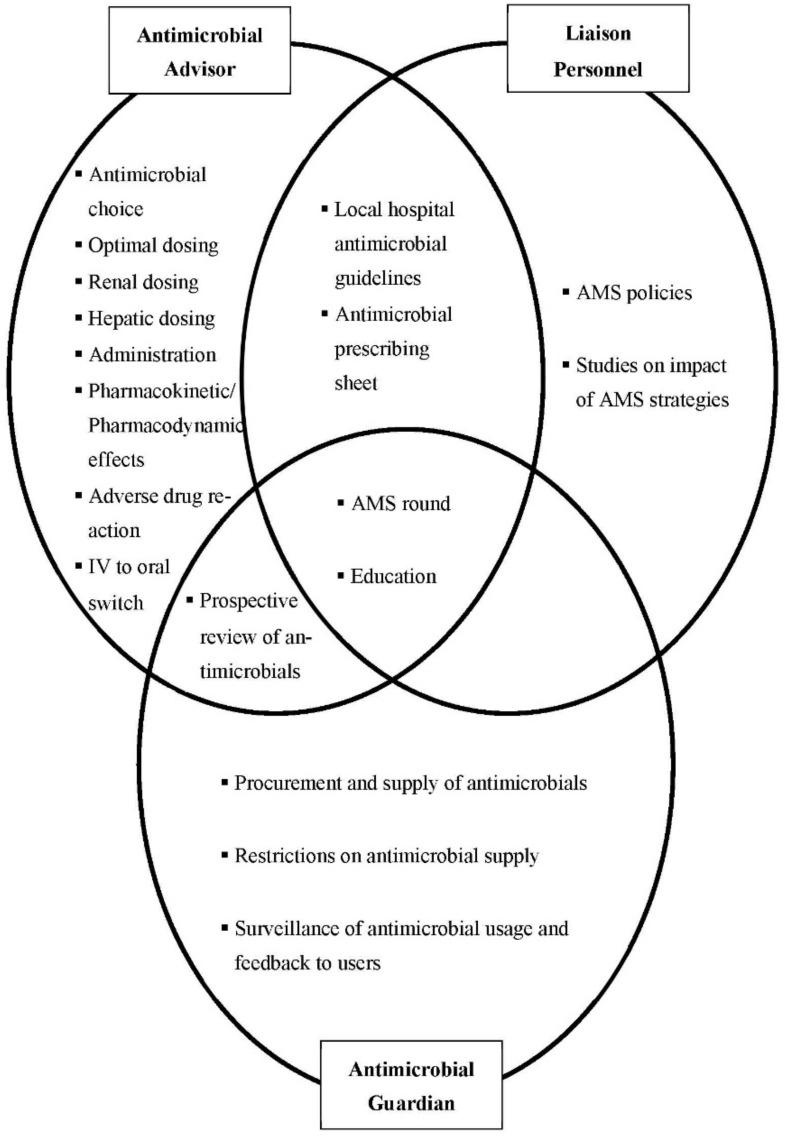
The interplay of pharmacists’ roles in the implementation of antimicrobial stewardship (AMS) strategies.

Pharmacists’ functions were categorized as antimicrobial advisors, antimicrobial guardians and liaison-personnel. Figure 1 summarizes the AMS strategies implemented, which draw on a single function or an interplay of pharmacists’ diverse functions.

Respondents described pharmacists’ advisory roles in providing evidence-based recommendations to physicians. Advising on antimicrobial choices, dosing, administration, adverse drug reactions and intravenous to oral switch were viewed as some of the core elements of pharmacists’ advisory roles.

*“…clinical pharmacists are the ones who really go into evidence-based [recommendations], and always intervene and recommend to the doctors on how to give appropriate antibiotics to all the patients according to accessibility and the local antibiogram.”*

(Respondent 7, university/state/major specialist hospital, 3 years of experience in AMS)

Pharmacists were also perceived to be apt guardians of antimicrobials, and their roles in this regard included exercising restriction strategies on antimicrobial supply and performing prospective reviews of antimicrobial orders. On top of the usual formulary restrictions, most respondents reported that additional review forms, for example carbapenem 72 h review forms, were introduced by pharmacists on behalf of the AMS team.

The surveillance and feedback on antimicrobial usage was also perceived as part of pharmacists’ functions as antimicrobial guardians. A few respondents reported taking additional initiatives to produce monthly surveillance reports to enable more timely feedback and to reduce the delay in any rectification strategies in response to the increase in antimicrobial usage.

In addition, respondents often identified pharmacists as liaison personnel in the AMS team who coordinate AMS-related activities and strategies. For example, they described their roles in liaising with department representatives during the development of local hospital guidelines and AMS policies:

*“…after the launch of our National Antibiotic Guidelines 2014, we extracted the data and divided it based on department, and then we assigned pharmacists to each department to sit down with the representatives from each department and microbiologists to go through the National Antibiotic Guidelines and see whether they agree to it or not. And if they don’t agree, to revise it based on evidence…after everyone has a consensus and agreement, we (pharmacists) compile and publish it as hospital antibiotic guidelines.”*

(Respondent 15, university/state/major specialist hospital, 1 year of experience in AMS)

Respondents also described pharmacists’ involvement in activities, such as AMS rounds and educational events, which exemplified the interplay of the three functions described above (Figure 1). For AMS rounds, respondents described how pharmacists identify patients from the antibiotic restriction and prospective review lists, and liaise with all responsible parties for the justification of antibiotic usage. Pharmacists’ roles in liaising with various quarters to organize educational activities for both the public and other healthcare professionals were also frequently highlighted. In these events, pharmacists educate on the judicious use of antimicrobials; and also explain regarding the implementation of antibiotic restriction and prospective review strategies.

#### 3.1.2. Visions on Role Expansion

Respondents envisaged involving and empowering more clinical pharmacists in the wards, especially those who are not directly involved in AMS teams, to incorporate AMS principles into their daily practices:

*“…we are trying to educate the ward pharmacists that anyone can do AMS and has to do AMS every day. So, it’s not just one AMS pharmacist doing AMS-related things… it doesn’t have to be something bombastic, something big. If [it is] just a matter of dose optimization, it is [also] considered AMS.”*

(Respondent 15, university/state/major specialist hospital, 1 year of experience in AMS)

When asked regarding the feasibility of clinical pharmacists having their own AMS rounds to increase the frequency of these rounds, in light of the busy schedules of infectious disease physicians, one respondent responded confidently:

*“Yes, I think it can be done…It is good and it does work because I think …if you really do work as a clinical pharmacist, we are more knowledgeable in antibiotics compared to doctors.”*

(Respondent 7, university/state/major specialist hospital, 3 years of experience in AMS)

However, most respondents still believed that pharmacists require support from doctors and stressed the importance of a team approach:

*“…our training is not the same as ID (infectious disease) physicians; they have that insight on certain pathophysiology and diagnosis… I would say for [pharmacists], we still need to be supported by ID physicians because they can help [us] to see certain different aspects… We can complement one another… I see it will be better to have a team approach.”*

(Respondent 5, university/state/major specialist hospital, 4 years of experience in AMS)

#### 3.1.3. Impact of Pharmacists’ Involvement in AMS

Respondents shared many positive impacts of pharmacists’ involvement in AMS, including a reduction in the inappropriate use of broad-spectrum antimicrobials and improved compliance with principles of good antimicrobial therapy. Respondents also identified many AMS strategies that acted as triggers to remind physicians to uphold the principles of judicious antimicrobial use, including AMS rounds, forms designed for antimicrobial review, and even the presence of pharmacists in the ward itself:

*“…by frequently having AMS rounds…the doctors [are] alert with their antibiotic orders, especially before the round, they will make sure everything is done (correctly) before we go for [the] antibiotic round.”*

(Respondent 16, university/state/major specialist hospital, 3 years of experience in AMS)

Importantly, many respondents also believed that their involvement in AMS helped to portray a better professional image of pharmacists among patients, as well as other healthcare professionals.

### 3.2. Barriers and Facilitators to Pharmacists’ Roles in the Implementation of AMS Strategies

Resources and attitudes are two sub-themes that emerged under the barriers and facilitators to pharmacists’ roles in the implementation of AMS strategies.

#### 3.2.1. Resources

The lack of resources in terms of training, manpower, time and facilities, were identified as some of the barriers that hospital pharmacists faced in the implementation of AMS strategies. The lack of specialized AMS training for pharmacists was a frequently identified barrier, whereby most respondents viewed the current one-day training course based on seminars to be insufficient:

*“Our training consists of talks rather than an attachment. I think for this to be successful, every AMS pharmacist must go through specialized training… talks and real-life [or] practical is different… for example myself… I have been attending talks, I have been reading online; I have been doing some learning but I have never ever been trained in a proper manner. So, I think because of that, we are not confident enough… we cannot, like, confidently suggest anything.”*

(Respondent 9, minor specialist/non-specialist hospital, 1 year of experience in AMS)

Some respondents who were relatively new to AMS described looking forward to a proposed training program for AMS pharmacists and attachment programs in hospitals with established AMS teams. In contrast, some respondents from larger hospitals with more established AMS teams looked at other countries for new ideas to improve their roles in patients’ care, such as pharmacy residency programs in Infectious Diseases.

Insufficient full-time AMS pharmacists, ID physicians and specialists, as well as time constraints related to pharmacists’ workload, were often cited by respondents as barriers to implementing their roles. The lack of full-time AMS pharmacists was perceived to hinder the progress of many AMS strategies and plans. All respondents, except for one, identified themselves as part-time AMS pharmacists and regarded that being a full-time AMS pharmacist would enable them to better focus on AMS-related activities.

*“One very big barrier is we hold multiple portfolios…so basically, I have to wear different hats, to read up different things. Then, we are also involved in teaching, meetings, and certain hospital quality-based work. So, that takes up a lot of time…”*

(Respondent 5, university/state/major specialist hospital, 4 years of experience in AMS)

In addition to the lack of full-time pharmacists, the lack of ID specialists was also cited as one of the reasons AMS rounds were not in place or not conducted on a regular basis, as they were often seen as the rightful leaders of AMS teams.

In terms of facilities, respondents from hospitals with information technology applications, such as the Total Hospital Information System which caters for all clinical and administrative activities in a hospital, described its usefulness in the daily execution of AMS activities. This included surveillance of antimicrobial usage, audit of high antimicrobial users and case identification for AMS rounds:

*“…the IT system has simplified the screening of patients because we can screen for culture and sensitivity results at the same time and it speeds up the screening process.”*

(Respondent 1, university/state/major specialist hospital, 4 years of experience in AMS)

Many respondents also believed that formal recognition of AMS committees by hospital administrators could facilitate the work process in implementing AMS strategies:

*“…because [the committee] becomes part of the administrative structure… that helped to ensure that some of the work through this antimicrobial stewardship committee will be recognized as a job worth doing…”*

(Respondent 5, university/state/major specialist hospital, 4 years of experience in AMS)

Administrative support from the hospital chief pharmacist was perceived by respondents to be vital in preventing the channeling of manpower away from AMS-related activities to other functions viewed as more critical. The chief pharmacist was also perceived to be influential in ensuring pharmacists from all units are equally exposed to AMS-related activities.

#### 3.2.2. Attitudes

The commitment of various parties to uphold the principles of AMS, including that of pharmacists, other healthcare professionals in the AMS team and non-AMS team members, was perceived to be an important factor influencing pharmacists’ roles in the implementation of AMS strategies. Respondents regarded their own commitment to continuous self-learning as a facilitator in enhancing AMS-related activities. Respondents, particularly those from the minor specialist or non-specialist hospitals, felt that some AMS team members were unwilling to commit their time to AMS-related activities. Many respondents also found commitment from non-AMS team members to be lacking, and cited examples such as absences from AMS-related discussions and meetings to discuss antimicrobial usage surveillance reports.

In terms of antimicrobial supply restriction strategies, respondents commented that pharmacists often have to deal with poor compliance with review forms, including incompletely filled out forms and a low return rate of forms. Respondents highlighted that because of poor compliance, restriction strategies were difficult to implement in the wards without clinical pharmacists. One respondent perceived pharmacists’ perseverance as a facilitator to ensure the sustainability of any restriction strategies, as the turnover rate of healthcare professionals is high and they would always need constant reminders to comply with restriction strategies.

## 4. Discussion

This study provided valuable insights into Malaysian hospital pharmacists’ roles in the implementation of AMS strategies in different public hospital categories, as well as the barriers and facilitators pharmacists faced in their role implementation.

In general, respondents perceived that pharmacists undertake most of the roles outlined for them in the Malaysian AMS protocol [16], and contribute significantly towards the implementation of the core strategies set out in the protocol. Pharmacists’ roles as antimicrobial guardians through the surveillance of antimicrobial use was a role most frequently described by the respondents, most likely because this function is monitored centrally by the Ministry of Health Pharmacy Practice Division for a national benchmarking process. The Infectious Diseases Society of America (IDSA) also endorsed feedback on surveillance data as a core element of an AMS program [23]. The pivotal utility of drug use evaluation performed by pharmacists was further highlighted in a study in Australia, whereby insufficient feedback on antimicrobial use was perceived as a barrier to effective AMS implementation [24]. Moreover, the importance of pharmacists’ antimicrobial guardian roles in the restriction and prospective review of antimicrobial supply is supported by a Cochrane review, which highlighted that such interventions improved compliance with antibiotic prescribing policies compared to no intervention [25].

The instrumental roles of pharmacists in liaising with other healthcare professionals in the development of local antimicrobial guidelines and advising on compliance with the guidelines, as reported by our respondents, are also supported by several studies [26,27]. The study by Al-eidan and colleagues demonstrated that the involvement of pharmacists in a multi-disciplinary team effort to encourage the use of a treatment protocol to manage community-acquired lower respiratory tract infections has resulted in many positive outcomes, including significant reductions in the length of hospital stay and healthcare costs [27]. Dean and colleagues reported a significant reduction in 30-day mortality for hospitalized patients when a guideline jointly developed by pharmacists and other healthcare professionals was implemented [26].

Our study highlighted pharmacists’ vision of expanding the practice of AMS strategies to all clinical pharmacists. This is in line with the recent change in the United States from a traditional AMS model led by an infectious disease (ID) physician and an ID-trained pharmacist, to a new model which advocates a team-based approach and empowers more generalist clinical pharmacists to be involved in AMS [28]. The new model involving generalist clinical pharmacists is seen to be more efficient and cost-effective, because pharmacists can monitor the patients as part of their daily routine [29]. Recently, hospitals in the United States have been moving towards empowering clinical pharmacists to authorize antimicrobial prescriptions which were traditionally authorized by ID physicians, and only cases disputed by physicians will be referred to the AMS team [28]. Both these traditional and new AMS models have their pros and cons and currently, there is still insufficient evidence to support either model [29].

While the focus of our respondents remained on the management of antimicrobials, pharmacists in other countries have shifted theirs to more holistic management of infections, rather than just focusing on antimicrobial prescribing. For example, one study in the United States described a pharmacist-initiated, health informatics-supported strategy that resulted in improvements in the quality of care for patients with *Staphylococcus aureus* bacteremia (SAB) [30]. AMS pharmacists in the United Kingdom concentrated on providing more convenient care for patients with Outpatient Parenteral Antibiotic Therapy (OPAT) [31]. In the local setting, there are only a few major specialist hospitals offering OPAT. This demonstrated that there is still room available for role expansion of local pharmacists in the design and implementation of AMS strategies.

Our findings showed that the lack of structured training was perceived to be a barrier to pharmacists’ roles in the implementation of AMS strategies. In the United States, training for AMS pharmacists involves post-graduate residency programs and board certification [18]. In the United Kingdom, an expert professional curriculum has been developed to train new AMS pharmacists [32]. The development of a comprehensive AMS training program in Malaysia should be accelerated to cater to local pharmacists’ needs. However, it should be acknowledged that, even with a structured training program, the availability of training might still be limited by the lack of funding and teaching support [18]. One of the possible strategies to address this is to adopt the “train the trainer” model currently used by leading AMS institutions in the United States [28]. This study further supports the need for a similar model, which is currently developed by the Pharmaceutical Services Division, Ministry of Health Malaysia.

The lack of manpower and full-time AMS pharmacists are obvious barriers representing a significant deviation from the recommendation of the Malaysian AMS protocol [16]. This predicament is also shared by many developed countries [17,18]. A survey on hospitals in England revealed that only 16% of the pharmacist posts were dedicated to full-time AMS work [17]. This reiterated that the lack of manpower is not an issue that can be resolved easily, even in countries with higher resources. Alternative strategies should be investigated, including the new AMS model described above that was adopted by the United States to empower more ward and clinical pharmacists to practice AMS [18]. The important element to note here is that appropriate training should be given prior to such role delegations.

The incorporation of an information technology system into the implementation of AMS strategies had been reported as a facilitator to pharmacists’ roles, especially in the United States [28,33]. This view, however, was not described by all respondents in our study. This is because not all public hospitals have access to applications such as the Total Hospital Information System (THIS), and the alternative Pharmacy Information System (PhIS) has not been integrated with computerized physician order entry (CPOE). Hence, many of the health informatics-supported interventions may not be feasible locally.

Our study did not show any inclination towards which AMS strategies are more likely to be adopted by larger hospitals (i.e., university, state and major specialist hospitals) as compared to minor specialist and non-specialist hospitals. However, pharmacists’ roles in the implementation of AMS strategies may be more frequently hindered in minor specialist and non-specialist hospitals due to the compounding effects of multiple barriers, compared with their counterparts in larger hospitals. ID physicians were not available in minor specialist and non-specialist hospitals by default of their hospital categories, and facilities to support good antimicrobial prescribing practices were more likely to be lacking. Our findings suggested that the lack of commitment among doctors and other healthcare professionals towards AMS was more frequently highlighted among respondents from minor specialists and non-specialist hospitals, which are usually located in rural areas. This is in concordance with the findings of a qualitative study in Australia, citing that continuous rotation of doctors in more rural hospitals complicated the implementation of AMS activities [24]. Motivated clinical champions can help to enact change through various educational strategies [34,35]. Pharmacists in minor specialist and non-specialist hospitals should perhaps consider taking up this challenge to improve the implementation of AMS strategies in their hospitals.

There are a number of limitations to this study. The results of this study may not be generalized to represent the views of AMS pharmacists in the country, as it was conducted in two states which have a larger number of major specialist hospitals compared to others [36]. Another point to note is that all respondents were AMS pharmacists from a clinical pharmacy background, hence their voices may not represent the views of pharmacists from different pharmacy units. The number of years of AMS experience of the respondents may have influenced their views, hence their views should be interpreted with this consideration in mind. Future research may expand on the current study to include the views of AMS pharmacists from other parts of the country. As AMS is a multidisciplinary effort, it will be interesting to also look into the views of other healthcare professionals on the challenges they are facing to ensure the prudent use of antibiotics.

## 5. Conclusions

This study has highlighted the important roles that public hospital pharmacists play in the AMS team, based on their functions as antimicrobial advisors, antimicrobial guardians and liaison personnel. Despite that, respondents acknowledged that there is still room for future role improvement and expansion. Further research is needed into strategies to facilitate role expansion, and on the support required by hospital pharmacists to overcome the barriers that have been identified in the implementation of AMS strategies.

## Figures and Tables

**Table 1:** Respondents’ demographic (*n* = 16).

Respondent Number	Gender	Years of Experience in AMS at Current Hospital	Current Position	Hospital Category
1	Female	4 years	Ward pharmacist	University/state/major specialist
2	Female	1 year	Ward pharmacist	University/state/major specialist
3	Female	1 year	Ward pharmacist	Minor specialist/non-specialist
4	Female	4 years	Ward pharmacist	University/state/major specialist
5	Female	4 years	Ward pharmacist	University/state/major specialist
6	Female	2 years	Ward pharmacist	University/state/major specialist
7	Female	3 years	Ward pharmacist	University/state/major specialist
8	Female	3 years	Ward pharmacist	Minor specialist/non-specialist
9	Male	1 year	Ward pharmacist	Minor specialist/non-specialist
10	Female	1 year	Ward pharmacist	University/state/major specialist
11	Female	1 year	Ward pharmacist	University/state/major specialist
12	Female	3 years	Ward pharmacist	University/state/major specialist
13	Female	1 year	Ward pharmacist	Minor specialist/non-specialist
14	Female	2 years	Ward pharmacist	University/state/major specialist
15	Female	1 year	Ward pharmacist	University/state/major specialist
16	Male	3 years	Ward pharmacist	University/state/major specialist

## Data Availability

Not applicable.
